# The Effect of Eight-Week Sprint Interval Training on Aerobic Performance of Elite Badminton Players

**DOI:** 10.3390/ijerph18020638

**Published:** 2021-01-13

**Authors:** Haochong Liu, Bo Leng, Qian Li, Ye Liu, Dapeng Bao, Yixiong Cui

**Affiliations:** 1 China Institute of Sport and Health Science, Beijing Sport University, Beijing 100084, China; liuhaochong1011@gmail.com (H.L.); olliejanessatdu62@gmail.com (Y.L.); 2 Sports Coaching College, Beijing Sport University, Beijing 100084, China; lengbo@bsu.edu.cn (B.L.); 2020110023@bsu.edu.cn (Q.L.); 3 AI Sports Engineering Lab, School of Sports Engineering, Beijing Sport University, Beijing 100084, China

**Keywords:** interval training, badminton, aerobic, repeated sprint, testing

## Abstract

This study was aimed to: (1) investigate the effects of physiological functions of sprint interval training (SIT) on the aerobic capacity of elite badminton players; and (2) explore the potential mechanisms of oxygen uptake, transport and recovery within the process. Thirty-two elite badminton players volunteered to participate and were randomly divided into experimental (Male-SIT and Female-SIT group) and control groups (Male-CON and Female-CON) within each gender. During a total of eight weeks, SIT group performed three times of SIT training per week, including two power bike trainings and one multi-ball training, while the CON group undertook two Fartlek runs and one regular multi-ball training. The distance of YO-YO IR2 test (which evaluates player’s ability to recover between high intensity intermittent exercises) for Male-SIT and Female-SIT groups increased from 1083.0 ± 205.8 m to 1217.5 ± 190.5 m, and from 725 ± 132.9 m to 840 ± 126.5 m (*p* < 0.05), respectively, which were significantly higher than both CON groups (*p* < 0.05). For the Male-SIT group, the ventilatory anaerobic threshold and ventilatory anaerobic threshold in percentage of VO_2_max significantly increased from 3088.4 ± 450.9 mL/min to 3665.3 ± 263.5 mL/min (*p* < 0.05),and from 74 ± 10% to 85 ± 3% (*p* < 0.05) after the intervention, and the increases were significantly higher than the Male-CON group (*p* < 0.05); for the Female-SIT group, the ventilatory anaerobic threshold and ventilatory anaerobic threshold in percentage of VO_2_max were significantly elevated from 1940.1 ± 112.8 mL/min to 2176.9 ± 78.6 mL/min, and from 75 ± 4% to 82 ± 4% (*p* < 0.05) after the intervention, which also were significantly higher than those of the Female-CON group (*p* < 0.05). Finally, the lactate clearance rate was raised from 13 ± 3% to 21 ± 4% (*p* < 0.05) and from 21 ± 5% to 27 ± 4% for both Male-SIT and Female-SIT groups when compared to the pre-test, and this increase was significantly higher than the control groups (*p* < 0.05). As a training method, SIT could substantially improve maximum aerobic capacity and aerobic recovery ability by improving the oxygen uptake and delivery, thus enhancing their rapid repeated sprinting ability.

## 1. Introduction

Badminton is a fast and dynamic sport, which has high requirements for the player’s rapid reaction, fast action and high-speed hitting ability. Studies have shown that there are on average 5–9 strokes in badminton games [1]. Due to its fastball speed, high swing frequency and short interval time, badminton requires players to mainly compete with fast running, sudden acceleration, abrupt stop, change of direction and continuously high intensity of multiple rallies, which requires a player’s well-developed aerobic endurance [2,3]. Due to the influence of players’ strength level, badminton competition is easy to form a multi-round competition, which requires higher aerobic working capacity. Particularly, a relevant study has indicated that badminton players usually reach an average heart rate of over 90% of their HRmax during competitive games, which is demanding to both aerobic and anaerobic systems: 60–70% on the aerobic system and 30% on the anaerobic system, with a greater demand on alactic metabolism [3].

Previous research [3,4] has shown that various physiological parameters had a strong correlation with badminton performance. Particularly, aerobic capacity and intermittent exercise performance are positively correlated, involving VO_2_max, lactate/anaerobic threshold and running efficiency [5]. However, practically, due to the limited training time, the traditional long-period aerobic endurance training would not be the most suitable modality for the actual needs of current competition. Therefore, it is essential to explore time-efficient and badminton-specific fitness training programs.

As one of the advocated alternatives to traditional continuous aerobic training, Sprint Interval Training (SIT) is a training approach that asks athletes to complete the required actions at maximum effort in a short period of time, and takes active rest with limited recovery time between two sets of training. Meanwhile, as the active rest often lasts only 3–5 min between multiple short and full sprint training, SIT can effectively improve the performance of athletes in intermittent sports with substantially lower overall training volume [6].

In recent years, there have been many studies on the training effect of SIT on sports performance in other intermittent sports such as soccer, basketball, volleyball and field hockey [7]. Among them, Buchan [8] and Bayati et al. [9] conducted a 6-week 30-s full-speed sprint running and rowing training experiment. Each training was carried out in 4–6 rounds, with 4 min of low-intensity activities serving as an active rest interval between groups. The results showed that the maximum oxygen uptake, peak power, average power and aerobic capacity significantly improved compared with those of the control group. Meanwhile, the study by Jone et al. [10] proved that field hockey players’ muscle oxygenation kinetics and performance during the 30–15 intermittent fitness test (30-s shuttle runs with 15-s passive recovery) were significantly improved after 6-week of Sprint Interval Cycling. Additionally, Burgomaster [11] and Gibala et al. [12] also conducted similar comparative experiments between traditional endurance training and SIT on young healthy individuals. Their results showed that the SIT group shortened the training time by about 80%, and the participants’ aerobic capacity was significantly improved.

Nonetheless, currently, there are few attempts to investigate the application of SIT to badminton training. This study was, therefore aimed to explore the effect of SIT on improving players’ aerobic capacity, as well as the mechanism of oxygen uptake and transport, by testing the changes of badminton players’ rapid and repeated sprinting ability and related aerobic capacity parameters before and after 8 weeks of SIT. It was hypothesized that such training would induce greater improvement in before-mentioned parameters compared to traditional continuous aerobic training.

## 2. Materials and Methods

### 2.1. Participants

Thirty-two elite players from who had played in or beyond the semi-finals of Badminton Championship at National level volunteered to participate in the study. There were sixteen male and female players, respectively, and they were randomly divided into male Sprint Interval Training (SIT) group (*n* = 8) and control (CON) group (*n* = 8), and female SIT group (*n* = 8) and CON group (*n* = 8). Detailed information about different groups can be found in Table 1. All subjects were in good health and had no severe injuries during the last six months before the study. Prior to the formal experiment and test, the nature and possible risks were explained to the participants, and they provided their written informed consent to participate. The tests were conducted at least 48 h after competitive match or heavy training session. The subjects participated in all the training sessions as well as pre- and post- training tests. All procedures were approved by Research Ethics Committee of Beijing Sport University (Approval number: 2020008H). All procedures were conducted in accordance with the Declaration of Helsinki.

### 2.2. Procedures

For eight weeks, the CON group followed previous training routines of two Fartlek running sessions and one regular multi-ball feeding training per week, which was a traditionally employed aerobic training protocol for these badminton players. Meanwhile, the SIT group carried out sprint interval training three times a week, including two power bicycle training sessions with a Monark 894E exercise bike (Monark Exercise AB, Vansbro, Sweden), which has high reliability of weight loading for anaerobic testing or training [13], and one SIT-specific multi-ball training session. Pedaling is a closed-chain exercise, and is relatively easier for the players to acquire correct technique and to achieve expected training effect from an injury-prevention perspective. Moreover, it is practically applicable to indoor badminton courts training during winter or in bad weather condition. The training intervention was designed and modified based on the previous literature [10,14,15]. The detailed training plan and description are shown in Table 2.

The Polar Team2 System (Polar Electro Oy, Kemple, Finland) was used to monitor the heart rate of each player throughout each training session, with data later extracted from custom-specific software (Polar Team2, Electro Oy, Kemple, Finland), in order to obtain maximum heart rate (HRmax), time spent in each HRmax% zone and Training impulse (TRIMP). TRIMP takes into account the training duration and intensity at the same time, and reflects the comprehensive effect of training on the internal and external load of the athlete’s body, as well as the load of medium and high intensity training. The method to determine the athlete’s TRIMP in the current study is based on the formula proposed by Edwards [16], where the time in each HRmax% zone is multiplied by the corresponding weighting factor for that zone and the results summated (see Table 3 for detailed description of the zone and factors). The HRmax of each player was established using the peak value recorded by the monitoring system during the training.

### 2.3. Test Program

Before and after 8 weeks of training, four groups all participated in a set of testing, which included YO-YO IR2 intermittent recovery test, analysis of the increasing load gas metabolism and lactate clearance rate test.

#### 2.3.1. YO-YO Intermittent Recovery Test Level 2 (YO-YO IR2 Test)

Speed endurance level is generally reflected by short bursts of repetitive sprints (RS), which requires subjects to try their best to accomplish the fastest speed in each repetitive sprint, and this ability is generally evaluated via the YO-YO IR2 test during field-test [6]. The test is based on increasing and intermittent load protocol, and evaluates player’s ability to recover between high intensity intermittent exercises. Moreover, it has been proven to validly monitor training effects [19].

After dynamic warm-up, players perform a combination of running to and fro on a 20 m course and a 10-s interval of active rest after 40 m, and players quit the test when the subjective exhaustion occurs or when they drop behind the required pace or make one of the errors listed below for a second time:

(i) does not come to a complete stop before starting the next 40 m run;
(ii) starts the run before the audio signal;
(iii) does not reach/either line before the audio signal;
(iv) turns at the 20 m mark without touching or crossing the line (therefore running short).

The starting speed starts at 13 km/h, and increases to 15 km/h, 16 km/h, and then increases by 0.5 km/h thereafter. The final running distance is then recorded. The speed of each bout is controlled by an audio recorder. All subjects were familiarized with the test within a one-minute trial.

#### 2.3.2. Analysis of Increasing Load of Gas Metabolism and Test of Lactate Clearance Rate

An incremental load test was performed using an incremental load treadmill (H/P Cosmos, Germany). Warm-up exercises should be performed for 5–10 min before each test. At the beginning of the test, the starting speed of the treadmill was set at 6 km/h, increasing by 1 km/h per minute, until 16 km/h, when the speed was stopped and the slope increased by 1.5% per minute, until the subject was exhausted. Relevant ventilation indicators such as maximum oxygen uptake (VO_2_max), ventilatory anaerobic threshold (VT-VO_2_) and ventilatory anaerobic threshold in percentage of VO_2_max (VT/VO_2_max) were measured using a gas metabolism analyzer (Max I, Physio-Dyne Instrument Corp., New York, USA). Among them, VT is determined according to the following criteria:

In the incremental load test, the VT value is determined (i) when the ratio of ventilation (VE) to carbon dioxide production (VCO2) shows a non-linear increase in the inflection point, and (ii) when the load intensity reaches a certain level, and the ratio of VE to oxygen consumption (VO_2_) increases sharply [20]. VT is determined by two independent investigators. When they are not coherent, and if the difference between the two selected results is remarkable, the value of VT needs to be determined again, while, if the difference could be overlooked, the average value is taken.

Next, in order to analyze the aerobic recovery speed of athletes after increasing load and to evaluate their recovery ability after aerobic exercise, blood samples were collected for rest (before testing with players being seated) and 0, 1, 3, 5, 7 and 10 min immediately after the increasing load test via a volume of 20 microliters of fingertip blood. The EKF Biosen s-line automatic blood lactate analyzer (EKF-diagnostic GmbH, Barleben, Germany) was used to measure blood lactate, with the results being later recorded with the lactate clearance rate being calculated using the following formula [21]:

$${\mathrm{LA}}_{10}\%\;=\frac{{\mathrm{LA}}_{max}-{\mathrm{LA}}_{10}}{{\mathrm{LA}}_{max}-{\mathrm{LA}}_{rest}}\times 100\%$$

where LA_10_% means the lactate clearance rate at 10 min after testing, LA*_max_* represents the peak lactic value after testing, LA_10_ is the lactate value at 10 min after testing and LA*_rest_* the value of lactate before testing.

### 2.4. Statistical Analysis

Experimental data were processed by SPSS statistical software package (version 23.0, Chicago, IL, USA); all test results before and after training were presented using the average ± standard deviation ($\bar{x}\;$± s). The normality of the tests results was checked before the subsequent analysis. A repetitive measure analysis of variance was then used to compare the within and between group difference in test outcomes for both genders, with the statistical significance level defined as *p* < 0.05. Pairwise differences and post hoc comparisons were tested with the Bonferroni post hoc test. Besides, the effect size (ES) was calculated using Cohen’s d to quantify the amount of change before and after each group of training and to reflect the comparison of training effects between SIT and CON groups based on the following scales: <0.2 trivial, 0.2–0.6 small, 0.6–1.2 moderate, 1.2–2.0 large and >2.0 very large [22].

## 3. Results

### 3.1. Training Intensity and Time Used During Training

Table 4 shows the descriptive statistics of heartrate and time within the 8-week training, and the results show that the average heart rate and maximum heart rate of both male and female SIT groups during training were significantly higher than those of the CON groups (*p* < 0.05). Moreover, the effective training time of the former was significantly less than that of the latter (*p* < 0.05).

**Figure 1:**
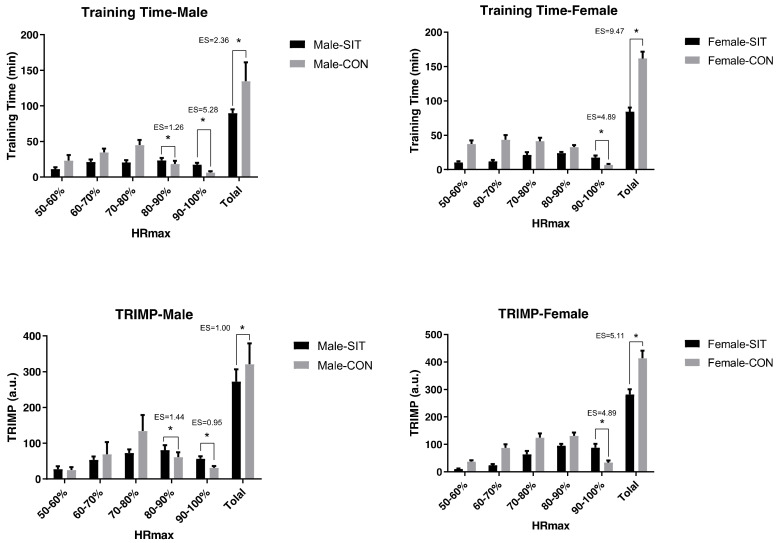
Comparisons of weekly effective training time and training impulse (TRIMP) between SIT and CON groups. Note: * Indicates a significant difference between SIT and CON group, *p* < 0.05.

During the 8-week training, the mean weekly effective training time (time spent within 50–100% HRmax zone) and TRIMP in the 80–100% HRmax intensity range of the Male-SIT group were significantly higher than those in the Male-CON group (*p* < 0.05), while the total weekly effective training time and TRIMP for the former were significantly lower than the latter (*p* < 0.05). As for female players, the average weekly effective training time and TRIMP in the 90–100%HRmax intensity range for the Female-SIT group were significantly higher than those in the Female-CON group (*p* < 0.05). However, in the intensity range of 80–90% HRmax, no differences were found between the F-SIT and F-CON groups. The overall effective training time and TRIMP for Female-SIT were significantly lower than those in Female-CON as well (*p* < 0.05), as is shown in Figure 1.

### 3.2. Comparisons of Testing Results Before and After Training Intervention

**Figure 2:**
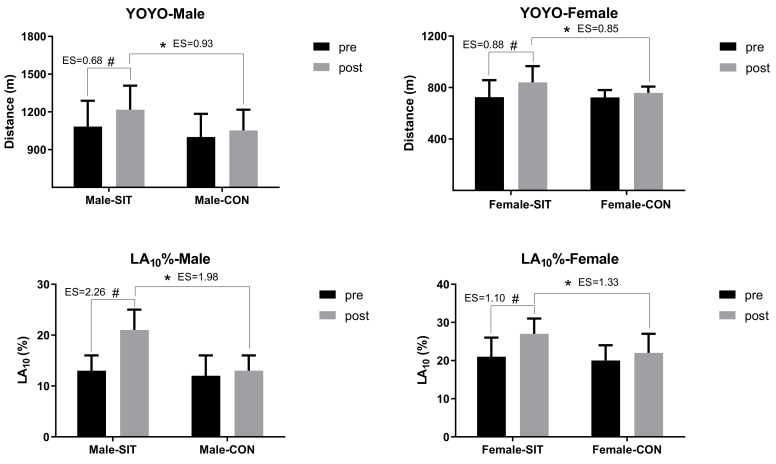
Within- and between-group differences in YO-YO Intermittent Recovery Test Level 2 (IR2) distance and lactate clearance rate for SIT and CON groups before and after intervention. Note: * Indicates significant difference between SIT and CON group, *p* < 0.05; # indicates significant within-group difference before and after intervention, *p* < 0.05; ES: effect size.

After training, the running distance of the YO-YO IR2 and the lactate clearance rate at 10 min after testing (LA_10_%) significantly increased in both the Male-SIT and the Female-SIT group (*p* < 0.05), and such improvement was significantly higher than that of the CON groups (*p* < 0.05), as is shown in Figure 2.

Meanwhile, as Table 5 demonstrates, VO_2_max, VT-VO_2_ and VT/VO_2_max for the SIT group significantly improved after the intervention (*p* < 0.05), and the improvement was significantly higher than that in the Male-CON group and Female-CON group (*p* < 0.05), as is shown in Table 4.

## 4. Discussion

This study was aimed to explore the effect of 8-weeks of SIT on the aerobic capacity of badminton players. The results showed that their performance in the YO-YO IR2 test, the lactate clearance rate, VO_2_max, VT-VO_2_ and VT/VO_2_max were significantly enhanced in a time-efficient manner, compared to the control group, which confirms the hypothesis of this research.

The badminton match is highly demanding to a player’s aerobic capacity due to the differences in individual physical fitness and the appearance of the new scoring model [4,5]. Under such situation, the competition rhythm is obviously accelerated and the proportion of multiple rallies is gradually increased, which forces players to endure longer periods of rapid and repeated accelerations and decelerations [23]. In the Male-SIT and Female-SIT groups, the time spent and TRIMP values in the 80–100% HRmax intensity interval accounted for the highest proportion, suggesting that SIT enables the body to complete multiple short-time and high-intensity outputs under the continuous incomplete recovery state, which is more in line with the current badminton competition characteristics and demands. In contrast to the previous training programs where all subjects routinely undertook Fartlek running, the 30-s SIT is a training mode closer to the maximum physiological load intensity of players, and the features of its time structure are also closer to the actual combat of badminton competition. It is an effective training program to improve a player’s aerobic capacity. Previously, it was reported that SIT could induce skeletal muscle metabolism, increase capillaries and mitochondrial proliferation, enhance oxidase activity and improve peripheral vascular function and peripheral fitness of skeletal muscle [24]. When training intensity exceeded 90%VO_2_max, SIT could simultaneously improve oxygen uptake and transport ability of the cardiopulmonary system and skeletal muscle [25]. Ermanno et al., found that intermittent exercise could activate the energy supply of the aerobic system in advance and reduce the proportion of the energy supply of the anaerobic system, thus delaying the generation of fatigue [25]. These changes in the body were physiological feedback for SIT. While the training improved the player’s ability to maintain high-intensity exercise for a long time in competition and training, their ability to recover from fast running could be improved, consequently achieving the goal of improving aerobic capacity.

Moreover, this study found that after 8 weeks of SIT, players’ VO_2_max, VT-VO_2_ and VT/VO_2_max increased significantly, implying that the proportion of exercise intensity lower than the anaerobic threshold for the body was increased under the same testing protocol. The time players take to enter the anaerobic glycolytic process would be postponed, thus reducing the consumption of glycogen. At the same time, the movement of the body would become more efficient, and eventually the maximum aerobic capacity of the players would be improved [26]. Previous studies showed that by inducing skeletal muscle metabolism, SIT could increase capillary proliferation, mitochondrial proliferation, enhance the activity and oxidation of glycolytic oxidase and improve peripheral vascular function and skeletal muscle peripheral adaptability [14,27]. Studies with similar schemes applied to the general population showed that the oxygen uptake and transport capacity were improved via a series of changes, such as increased capillary density and blood volume, decreased heart rate and increased stroke output, when the same exercise intensity was completed. At this time, the body showed certain adaptability. In practice, with the improvement of the body’s oxygen uptake ability, elite badminton players could prolong the time of oxygen supply and enter the hypoxemia state later in the competition, which could effectively improve their match performance during the competition. Besides, although some research showed certain discrepancies in results, we found that after the SIT, no significant increase in VO_2_max was indicated. It would be inferred that the effect of such modality would be conditioned by factors such as the level of training, whether the subjects undertake regular training and the body weight.

Aerobic recovery ability has a direct impact on players’ on-court performance. High-intensity and high-load activity during competition would produce physiological fatigue and large amount of lactate accumulation in the skeletal muscle. Changes in the internal responses of the body may cause players’ physical dysfunction and decline in athletic performance [28,29]. Therefore, rapid recovery ability is the key prerequisite for decent physical and technical performance during the competition. This study analyzed the changes in skeletal muscle’s oxygen recovery ability from both physiological and biochemical perspectives.

Blood lactate is one of the most commonly used biochemical indicators to detect the body fatigue recovery status [30], and the accumulation of lactate may indirectly lead to reduced performance, because the conversion of lactic acid to lactate releases H^+^ that leads to a metabolic acidosis with subsequent inhibition of glycolytic rate-limiting enzymes, lipolysis and contractility of the skeletal muscles [31]. From the results, it was shown that SIT performed at a higher level of intensity could positively influence the clearance of lactate after exercise, increasing intra-cellular alkali reserve and slowing the pH reduction in muscles, and delaying the onset of fatigue [21]. Consequently, players’ ability to recover from intermittent activities was enhanced and they would be better prepared for the next point and game [32]. In particular, at the final game and the last points of each game, each point would be ended with prolonged multiple-strokes and high-intensity movements, which might even last couple of minutes. Under such circumstances, possessing rapid aerobic recovery would become a key factor determining elite player’s aerobic endurance and technical-tactical performance in the next point [33]. In the study conducted by Jones et al. [10], near-infrared spectroscopy was used to measure muscle oxygenation of the vastus lateralis of elite women hockey players for SIT groups and endurance training groups. Their results showed that there were significant increases in tissue deoxyhaemoglobin + deoxymyoglobin (HHb + HMb) and tissue oxygenation (TSI%), and a significant decrease in tissue oxyhaemoglobin + oxymyoglobin (HbO2 + MbO2), which indicated ‘positive peripheral muscle oxygen adaptations’ occurring in response to SIT training. Moreover, existing literature also stated that the higher exercise intensity provided during SIT would increase the probability of favorable adaptations in both type one and type two fibers as opposed to the generally lower intensity of endurance training [34]. Although as a limitation, the current study was unable to measure blood saturation, it could be implied that the SIT protocol might promote the skeletal muscle oxidative capacity of badminton players after the training. Nonetheless, future studies should look into the changes of EPOC (excess post-exercise oxygen consumption), body temperature and ventilation to comprehensively verify the improvement in recovery after such intervention.

## 5. Conclusions

Eight-week SIT effectively improved the aerobic exercise capacity of elite badminton players, particularly considering oxygen uptake and recovery ability, and the adaptability of skeletal muscle to exercising load. Eventually, the rapidly repeated sprint ability and physical performance of players were enhanced. The study has provided evidence-based findings that as a time-efficient training alternative, SIT could be suitable to be included in the training routine for badminton players.

However, it is acknowledged that this study also has certain limitations. The technical and tactical performance was not considered, which might be another representative indicator of improved aerobic capacity. Moreover, anaerobic endurance training, strength training and functional training are also of vital importance for badminton players and their joint effect on aerobic training was not investigated within the current program. Future research is suggested to look into these aspects to better inform sport-specific training prescription.

## Figures and Tables

**Table 1:** Personal information of participating players.

Group	N	Age (year)	Height (cm)	Weight (kg)	Training Age (year)	HRmax (bpm)
Male-SIT	8	20.0 ± 1.3	179.6 ± 3.6	73.8 ± 6.9	12.1 ± 2.2	190.7 ± 8.8
Male-CON	8	21.5 ± 2.2	177.1 ± 7.1	72.4 ± 6.7	13.2 ± 3.2	191.8 ± 6.2
Female-SIT	8	20.5 ± 1.4	168.5 ± 4.2	62.6 ± 4.2	9.5 ± 1.2	181.9 ± 8.9
Female-CON	8	19.4 ± 1.5	168.2 ± 4.8	61.3 ± 4.2	9.8 ± 1.5	180.4 ± 8.5

Note: SIT = Sprint Interval Training; CON = Control; HRmax = maximum heart rate.

**Table 2:** Weekly training plan for two groups during the study.

Group	Monday	Wednesday	Friday
SIT	*SIT Cycling Training* 1–2 min 50 W cycling, prepare to 30 s cycling with full force, the load is 0.075/kg of weight individualized to each player’s body weight [17,18], between-group rest: 5 min 5 groups in total		*SIT-specific Multiple Balls Training* 30 s × 8 groups × 2 rounds of multi-ball training, intensity: > 90% HRmax between-group rest: 5 min between-round rest: 8 min
CON	*Traditional Training:* 40 min of Fartlek Run (Intensity: 65–79% HRmax)		*Traditional Multiple Ball Training:* 1 min × 4 groups × 2 rounds of continuous multi-ball training between-group rest: 5 min between-round rest: 8 min

**Table 3:** HRmax% zones and corresponding weighting factors.

Zone	Weighting Factor	HRmax%
I	1	50–60%
II	2	60–70%
III	3	70–80%
IV	4	80–90%
V	5	90–100%

**Table 4:** Intensity monitoring during training.

Group	N	Avg HR (bpm)	HRmax (bpm)	Total Training Time (min)	Effective Training Time (min)
Male-SIT	8	132.7 ± 7.3 *	190.7 ± 8.8 *	52.7 ± 4.1	19.8 ± 3.0 *
Male-CON	8	126.0 ± 10.2	169.8 ± 6.2	78.5 ± 4.5	40.2 ± 1.8
Female-SIT	8	134.1 ± 6.0 *	181.9 ± 8.9 *	52.7 ± 4.1	19.8 ± 3.0 *
Female-CON	8	115.4 ± 8.4	169.4 ± 8.5	78.5 ± 4.5	40.2 ± 1.8

Note: Values are expressed as means ± SD. * indicates significant difference between SIT and CON group, *p* < 0.05.

**Table 5:** Within- and between-group differences in gas metabolism analysis for SIT and CON groups before and after intervention.

Group		VO_2_max (mL/kg/min)	VT-VO_2_ (mL/min)	VT/VO_2_max (%)
Male-SIT	pre	56.8 ± 7.0	3088.4 ± 450.9	73.8 ± 9.7
	post	63.6 ± 4.7 ^#,^*	3665.3 ± 263.5 ^#,^*	84.8 ± 3.37 ^#,^*
	ES	1.14	1.56	1.51
Male-CON	pre	55.8 ± 8.0	2962.5 ± 743.4	80.2 ± 1.5
	post	57.7 ± 6.7	3004.1 ± 738.1	80.8 ± 2.3
	ES	0.26	0.06	0.31
Female-SIT	pre	42.5 ± 2.9	1940.1 ± 112.9	0.75 ± 0.04
	post	46.2 ± 3.0 *^,#^	2176.9 ± 78.6 *^,#^	0.82 ± 0.04 *^,#^
	ES	1.28	1.11	1.75
Female-CON	pre	42.9 ± 1.6	1930.7 ± 151.8	0.75 ± 0.06
	post	43.3 ± 2.1	2055.3 ± 160.7	0.78 ± 0.08
	ES	0.21	0.79	0.42

Note: * Indicates significant difference between SIT and CON group, *p* < 0.05; ^#^ indicates significant within-group difference before and after intervention, *p* < 0.05; ES: effect size.

## References

[1] Jan C., Petr S. (2020). Serve and Return in Badminton: Gender Differences of Elite Badminton Players. Int. J. Phys. Educ. Fit. Sports.

[2] Nhan D.T., Klyce W., Lee R.J. (2018). Epidemiological Patterns of Alternative Racquet-Sport Injuries in the United States, 1997–2016. Orthop. J. Sports Med..

[3] Phomsoupha M., Laffaye G. (2015). The science of badminton: Game characteristics, anthropometry, physiology, visual fitness and biomechanics. Sports Med..

[4] Laffaye G., Phomsoupha M., Dor F. (2015). Changes in the Game Characteristics of a Badminton Match: A Longitudinal Study through the Olympic Game Finals Analysis in Men’s Singles. J. Sports Sci. Med..

[5] Faude O., Meyer T., Rosenberger F., Fries M., Huber G., Kindermann W. (2007). Physiological characteristics of badminton match play. Eur. J. Appl. Physiol..

[6] Bangsbo J., Iaia F.M., Krustrup P. (2008). The Yo-Yo intermittent recovery test: A useful tool for evaluation of physical performance in intermittent sports. Sports Med..

[7] Kelly D.T., Tobin C., Egan B., McCarren A., O’Connor P.L., McCaffrey N., Moyna N.M. (2018). Comparison of Sprint Interval and Endurance Training in Team Sport Athletes. J. Strength Cond. Res..

[8] Buchan D.S., Ollis S., Young J.D., Thomas N.E., Cooper S.M., Tong T.K., Nie J., Malina R.M., Baker J.S. (2011). The effects of time and intensity of exercise on novel and established markers of CVD in adolescent youth. Am. J. Hum. Biol..

[9] Bayati M., Farzad B., Gharakhanlou R., Agha-Alinejad H. (2011). A practical model of low-volume high-intensity interval training induces performance and metabolic adaptations that resemble ‘all-out’ sprint interval training. J. Sports Sci. Med..

[10] Jones B., Hamilton D.K., Cooper C.E. (2015). Muscle Oxygen Changes following Sprint Interval Cycling Training in Elite Field Hockey Players. PLoS ONE.

[11] Burgomaster K.A., Heigenhauser G.J., Gibala M.J. (2006). Effect of short-term sprint interval training on human skeletal muscle carbohydrate metabolism during exercise and time-trial performance. J. Appl. Physiol..

[12] Gibala M.J., Little J.P., van Essen M., Wilkin G.P., Burgomaster K.A., Safdar A., Raha S., Tarnopolsky M.A. (2006). Short-term sprint interval versus traditional endurance training: Similar initial adaptations in human skeletal muscle and exercise performance. J. Physiol..

[13] Lunn W.R., Axtell R.S. (2019). Validity and Reliability of the Lode Excalibur Sport Cycle Ergometer for the Wingate Anaerobic Test. J. Strength Cond. Res..

[14] Gist N.H., Fedewa M.V., Dishman R.K., Cureton K.J. (2014). Sprint interval training effects on aerobic capacity: A systematic review and meta-analysis. Sports Med..

[15] Hostrup M., Gunnarsson T.P., Fiorenza M., Mørch K., Onslev J., Pedersen K.M., Bangsbo J. (2019). In-season adaptations to intense intermittent training and sprint interval training in sub-elite football players. Scand. J. Med. Sci. Sports.

[16] Edwards S. (1994). The Heart Rate Monitor Book. Med. Sci. Sports Exerc..

[17] Bar-Or O., Dotan R., Inbar O. (1977). A 30 second all-out ergometric test-its reliability and validity for anaerobic capacity. Isr. J. Med. Sci..

[18] Laurent C.M., Meyers M.C., Robinson C.A., Green J.M. (2007). Cross-validation of the 20-versus 30-s Wingate anaerobic test. Eur. J. Appl. Physiol..

[19] Schmitz B., Pfeifer C., Kreitz K., Borowski M., Faldum A., Brand S.M. (2018). The Yo-Yo Intermittent Tests: A Systematic Review and Structured Compendium of Test Results. Front. Physiol..

[20] Binder R.K., Wonisch M., Corra U., Cohen-Solal A., Vanhees L., Saner H., Schmid J.-P. (2008). Methodological approach to the first and second lactate threshold in incremental cardiopulmonary exercise testing. Eur. J. Cardiovasc. Prev. Rehabil..

[21] Wang J., Qiu J., Yi L., Hou Z., Benardot D., Cao W. (2019). Effect of sodium bicarbonate ingestion during 6 weeks of HIIT on anaerobic performance of college students. J. Int. Soc. Sports Nutr..

[22] Hopkins W., Marshall S., Batterham A., Hanin J. (2009). Progressive statistics for studies in sports medicine and exercise science. Med. Sci. Sports Exerc..

[23] Gomez M.A., Rivas F., Connor J.D., Leicht A.S. (2019). Performance Differences of Temporal Parameters and Point Outcome between Elite Men’s and Women’s Badminton Players According to Match-Related Contexts. Int. J. Environ. Res. Public Health.

[24] Buchheit M., Abbiss C.R., Peiffer J.J., Laursen P.B. (2012). Performance and physiological responses during a sprint interval training session: Relationships with muscle oxygenation and pulmonary oxygen uptake kinetics. Eur. J. Appl. Physiol..

[25] Koral J., Oranchuk D.J., Herrera R., Millet G.Y. (2018). Six Sessions of Sprint Interval Training Improves Running Performance in Trained Athletes. J. Strength Cond. Res..

[26] Connolly D.A. (2012). The anaerobic threshold: Over-valued or under-utilized? A novel concept to enhance lipid optimization!. Curr Opin. Clin. Nutr. Metab. Care.

[27] Phillips S.M., Sproule J., Turner A.P. (2011). Carbohydrate ingestion during team games exercise: Current knowledge and areas for future investigation. Sports Med..

[28] Meeusen R., Watson P., Hasegawa H., Roelands B., Piacentini M.F. (2006). Central fatigue: The serotonin hypothesis and beyond. Sports Med..

[29] Welsh R.S., Davis J.M., Burke J.R., Williams H.G. (2002). Carbohydrates and physical/mental performance during intermittent exercise to fatigue. Med. Sci. Sports Exerc..

[30] Robergs R.A., McNulty C.R., Minett G.M., Holland J., Trajano G. (2018). Lactate, not lactic acid, is produced by cellular cytosolic energy catabolism. Physiology.

[31] Menzies P., Menzies C., McIntyre L., Paterson P., Wilson J., Kemi O.J. (2010). Blood lactate clearance during active recovery after an intense running bout depends on the intensity of the active recovery. J. Sports Sci..

[32] Fukuoka Y., Iihoshi M., Nazunin J.T., Abe D., Fukuba Y. (2017). Dynamic Characteristics of Ventilatory and Gas Exchange during Sinusoidal Walking in Humans. PLoS ONE.

[33] Andersen L.L., Larsson B., Overgaard H., Aagaard P. (2007). Torque–velocity characteristics and contractile rate of force development in elite badminton players. Eur. J. Sport Sci..

[34] Hood D.A. (2001). Invited Review: Contractile activity-induced mitochondrial biogenesis in skeletal muscle. J. Appl. Physiol..

